# Effectiveness and safety of acupuncture combined with traditional Chinese medicine in the treatment of chronic prostatitis: A systematic review and meta-analysis

**DOI:** 10.1097/MD.0000000000028163

**Published:** 2021-12-10

**Authors:** Chenxi Li, Lei Xu, Xuyao Lin, Qingrui Li, Pule Ye, Lin Wu, Mingkai Wang, Lichao Li, Lanlan Li, Yue Zhang, Hua Li, Guozheng Qin

**Affiliations:** a Yunnan University of Chinese Medicine, Kunming, China; b The First Affiliated Hospital of Yunnan University of Chinese Medicine, Kunming, China; c Aerospace Central Hospital, Beijing, China; d Lincang Hospital of Traditional Chinese Medicine, Lincang, China.

**Keywords:** acupuncture, chronic prostatitis, efficacy, meta-analysis, safety, traditional Chinese medicine

## Abstract

**Objective:**

Chronic prostatitis (CP) is a common disease in the outpatient department of males and urology. Clinical studies have found that acupuncture combined with traditional Chinese medicine (TCM) has achieved good results in treating CP, but its efficacy and safety are not completely clear. This study aimed to investigate the efficacy and safety of acupuncture combined with TCM in the treatment of CP.

**Methods:**

Randomized controlled trials of acupuncture combined with TCM in treating CP were screened by searching PubMed, Embase, Cochrane Library, CNKI, etc. The retrieval time was from the database establishment date to March 31, 2021. The Cochrane Collaborative Risk Bias Assessment tool was used to evaluate literature's methodological quality of the literature. The RevMan5.4 software was used for the meta-analysis of outcome indicators. The TSA v0.9 software was used for sequential trial analysis (TSA) of effectiveness.

**Results:**

In this study, 19 related randomized controlled trial studies were included, with a total of 1831 cases. The results of the meta-analysis showed that acupuncture combined with TCM could significantly improve the clinical efficacy of CP (OR = 3.76, 95%CI: 2.82 to 5.02, *P* < .00001), reduce the total score of The National Institutes of Health chronic prostatitis symptom index (MD = −4.00, 95%CI: −4.67 to 3.33, *P* < .00001), and improve patients’ urination symptoms (MD = −1.10, 95%CI: −1.23 to −0.97, *P* < .00001), alleviated the pain symptoms of patients (MD = −2.38, 95%CI: −2.41 to −2.35, *P* < .00001), improved the quality of life of patients (MD = −1.69, 95%CI: −1.97 to −1.41, *P* < .00001), decreased the scores of TCM symptoms of patients (MD = −2.39, 95%CI: −3.45 to −1.33, *P* < .00001), and did not increase the adverse reactions of patients (MD = 1.09, 95%CI: 0.57 to 2.06, *P* = .8). The results of publication bias showed that this study was not affected by publication bias, and the conclusion was reliable. TSA showed that acupuncture combined with TCM was effective in treating CP.

**Conclusion:**

Acupuncture combined with TCM is safe and effective for alleviating CP. It can be used as an effective treatment for chronic prostatitis in the clinic.

**Registration number:** DOI 10.17605/OSF.IO/Z8FJM.

## 1 Introduction

Prostatitis is one of the common diseases in the outpatient department of andrology and urology. Its incidence is on the rise year by year, and about 10% to 14% of European and American men are affected by prostatitis.^[1]^ In China, the incidence of CP is higher than in other countries, about 6.0% to 32.9%,^[2–3]^ but only 5% to 10% of the patients are caused by bacterial infection.^[4–5]^ Chronic prostatitis/chronic pelvic pain syndrome (CP/CPPS) is clinically more common, accounting for more than 90% of patients with prostatitis. Characterized by chronic, recurrent pelvic pain or discomfort, CP/CPPS syndrome may last for more than 3 months, and is accompanied by sexual dysfunction and various urinary symptoms.^[6]^ CP/CPPS syndrome is closely associated with other systemic syndromes such as irritable bowel syndrome, fibromyalgia, cardiovascular disease, stress, depression, and anxiety.^[7]^ The quality of life of patients with CP/CPPS was comparable to that of patients with myocardial infarction or Crohn disease in terms of pain and deterioration of quality of life alone.^[8]^ Given that CP has a tremendous negative impact on men's mental and psychological aspects around the world, and even causes disharmony in family relations, actively seeking safe and effective drugs to prevent recurrence is a current research hotspot.

The etiology, pathogenesis, and pathophysiology of CP are currently diverse. Studies have shown that its occurrence may be related to unknown microbial infection, autoimmune abnormalities, oxidative stress, endocrine diseases, neurological diseases, and social psychology.^[9–10]^ Modern medical interventions for CP mainly include alpha-blockers, antibiotics, analgesics, and multimodal therapy.^[11–13]^ However, its therapeutic effect is limited, and there is currently no good strategy to relieve the symptoms of CP/CPPS. Anti-inflammatory drugs to treat CP are easy to cause serious side effects of gastrointestinal and cardiovascular diseases, and the efficacy is not significant. Meanwhile, the symptoms are easy to relapse. Non-drug treatments such as acupuncture are gaining traction. Until now, acupuncture was commonly used for CP and chronic pain relief in eastern countries.^[14]^ Recent studies have shown remarkable effects in reducing chronic pain and tissue fibrosis around the pelvic floor area.^[15–17]^ Studies also show that acupuncture could accelerate the disease central nervous system to produce endogenous opioid peptides and activate relevant receptors by stimulating related acupoints, which achieves peripheral analgesia. Besides, it could also achieve anti-inflammatory effects by promoting the B-EP levels in inflammatory tissue and serum.^[18–20]^ Traditional Chinese medicine (TCM) has been playing the role of treating diseases and saving people for thousands of years in China, which is the crystallization of the labor and wisdom of the Chinese nation. The principle of treating diseases is to correct the bias of diseases through different herbs’ medicinal properties. Because of its macroscopic, holistic, and flexible treatment principles, and unclear explanation of its microscopic principles, it has not been widely accepted by people in other countries, but its efficacy against many diseases is beyond doubt. China has used TCM to fight epidemics more than 300 times, bringing to the world the advanced concept of “vaccination and prevention of infectious diseases.” Tu Youyou discovery of artemisinin has saved the lives of millions of people and presented a great gift to the world. In the battle against SARS and novel coronavirus infection, when the world was at a loss, TCM stepped forward and played a vital role, which was favored and appreciated by many foreign friends. CP is a kind of refractory disease, and its pathogenesis has not been completely clear. TCM treatment of CP/CPPS has a profound theoretical basis and rich clinical experience.^[21]^ TCM mainly achieves therapeutic effects by stimulating the body's vital qi and regulating the balance of qi and blood.^[22]^ Modern studies have shown that the effective active ingredients in TCM can achieve the therapeutic purpose by improving the blood supply of peripheral blood vessels.^[19,20]^ Moreover, Chinese medicine can relieve the pain and improve the quality of life of patients with CP/CPPS, and these effects are persistent.^[19,23]^

Based on the macro perspective, acupuncture and TCM treat patients with different CP syndrome differentiation. The methods are flexible and diverse, with little toxic and side effects and no recurrence of patients’ symptoms, which reveals the advantages of their combined application in the treatment of CP. To evaluate the clinical efficacy and safety of acupuncture combined with TCM to treat CP, we will select randomized controlled trials of acupuncture combined with TCM in the treatment of CP through data search of major databases and conduct systematic evaluation and meta-analysis.

## 2 Method

This systematic review was performed in accordance with the Preferred Reporting Items for Systematic Review and Meta-Analysis statement. The study was registered on the Open Science Framework platform, DOI 10.17605/OSF.IO/Z8FJM.

### 2.1 Literature inclusion criteria

#### 2.1.1 Participants

Patients who were diagnosed with CP by andrologists or urologists in national public hospitals (as follows). In addition, there are no restrictions on region, nationality, race, etc.

1) Patients who meet the diagnostic criteria of CP in Western medicine.
2) Duration of disease longer than 3 months.
3) Aged 18 to 70 years old.
4) Patients who had received treatment before consultation could be included in the study after stopping medication for at least 2 weeks.
5) Patients who signed the informed consent.

#### 2.1.2 Intervention measures

Acupuncture combined with TCM (such as TCM prescriptions and proprietary Chinese medicine) was used in the treatment group, with no restrictions on the way of administration. The control group was treated with acupuncture, TCM, or placebo. Baseline data of the 2 groups were well comparable.

#### 2.1.3 Outcome indicators

Main outcome indicators included effective rate and incidence of adverse reactions; Secondary outcome measures included the total The National Institutes of Health chronic prostatitis symptom indexscore, pain symptom score, urination symptom score, quality of life score, TCM syndrome score, etc.

#### 2.1.4 Study type

Conformed to randomized controlled trial (RCT).

### 2.2 Literature exclusion criteria

1) Patients with severe heart, liver, and kidney dysfunction and brain organic diseases.
2) Clinical studies where complete data are not available.
3) Patients with a history of pelvic surgery.
4) Case report, case analysis, cross-sectional study, case–control study, cohort study, and other non-RCT research literature.
5) Men with abnormal development or genitourinary system infection.
6) Prostatic tuberculosis, benign prostatic hyperplasia, prostate cancer, and acute urethral syndrome.
7) The treatment group or the control group used or combined with the use of drugs not belonging to TCM.

### 2.3 Literature retrieval strategy

The primary databases retrieved included PubMed, Embase, Cochrane Library, CNKI, Wanfang, etc. The retrieval time is from the establishment to March 31, 2021. The English search terms were chronic prostatitis, chronic pain syndrome, acupuncture, acupoints, TCM, prescription, randomized, clinical trials, etc. Each search term is searched individually or jointly, and the references of the literature are screened. After reading the title, a retrospective search is carried out.

### 2.4 Data screening and data extraction

NoteExpress 3.3 software was used for literature management, and the retrieved literature was imported into it and the database was established. According to the inclusion and exclusion criteria and the purpose of the study, 3 researchers independently screened and extracted the data from the literature, discussed and dealt with the problems in data screening and data extraction, and checked the extraction and input result one by one. The literature with missing, inaccurate, or incomplete data was excluded. The general information of the included literature, the basic information of the research methods and the subjects, the intervention measures of the treatment group and the control group, the outcome indicators, and other materials and data were extracted. Primary outcome measures included efficacy and adverse events, while secondary outcome measures included total NIH-CPSI score, pain symptom score, urination symptom score, quality of life score, TCM syndrome score, etc. The multi-arm test was converted to the two-arm test, and the treatment group and the control group were screened according to the inclusion criteria, and the data were extracted.

### 2.5 Literature quality evaluation

The quality of the included literature was evaluated by referring to the “risk of bias” evaluation table entry of the Cochrane system evaluation standard. The risk of bias was evaluated by RevMan5.4 software, and a summary chart of the risk of bias was drawn. The evaluation process was carried out by 2 researchers alone, then summarized and discussed.

### 2.6 Data analysis

Meta-analysis was performed using RevMan5.4 software. The odds ratio (OR) was used as the effect size index for the dichotomous variables, and the Weighted Mean Difference was used as the effect size index for the continuous variables, with 95% confidence intervals (CI). *P* ≤ .05 indicated a statistically significant difference. *I*^2^ value was used to evaluate the heterogeneity: when *I*^2^ ≥ 50%, the heterogeneity was obvious, and a random-effects model was used to synthesize the heterogeneity. Subgroup analysis was used when necessary, or sensitivity analysis was conducted for studies with inconsistent inclusion results to explore the heterogeneity. When *I*^2^ ≤ 50%, heterogeneity was ignored and a fixed effect model was used. TSA V0.9 software was used for sequential analysis of the efficiency. RevMan5.4 software was used to analyze the publication bias of major outcome indicators. When the funnel plot was symmetrical on both sides, the possibility of publication bias between studies was considered low.

## 3 Results

### 3.1 Literature screening results

**Figure 1:**
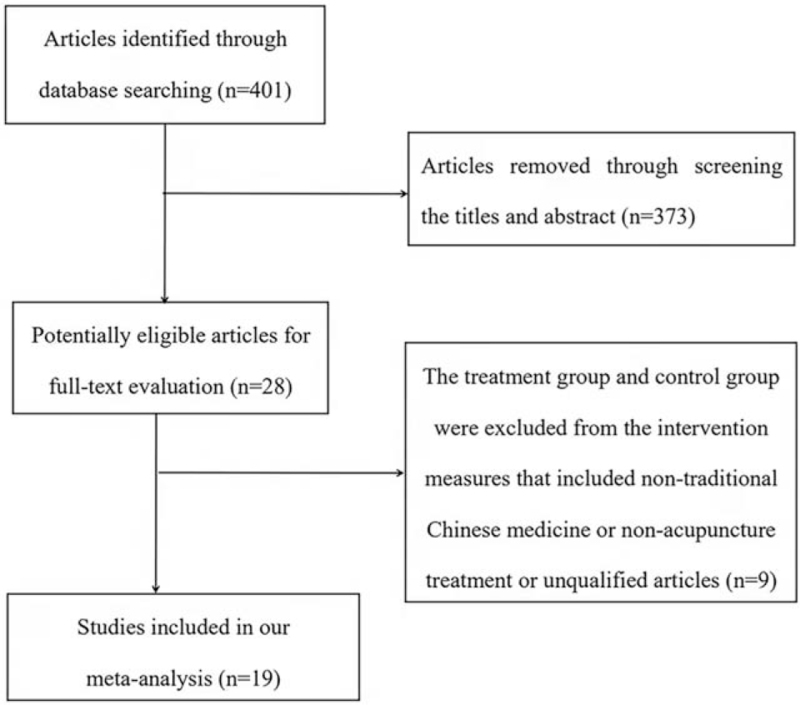
Literature screening flow chart.

The retrieval results were retrieved from the above 6 databases, and 401 citations were found. After reading the titles and abstracts, 373 of them were excluded, leaving 28 citations. After reading the full text, it was found that 9 RCTs in the treatment group or control group were combined with other medicine. After the exclusion, the remaining 19 RCTs met our inclusion criteria. The study flow chart is shown in Figure 1.

### 3.2 Characteristics of included trials

In this study, 19 relevant RCT studies were included, with a total of 1831 cases. Of these, 21 trials reported the primary outcome measure response rate, 7 trials reported the NIH-CPSI overall score, 7 trials reported the NIH-CPSI urination symptom score and pain symptom score, 9 trials mentioned the NIH-CPSI quality of life score, 4 trials reported the TCM syndrome score, and 4 trials reported adverse events. The basic characteristics of the included trials are shown in Table 1.

**Table 1:**
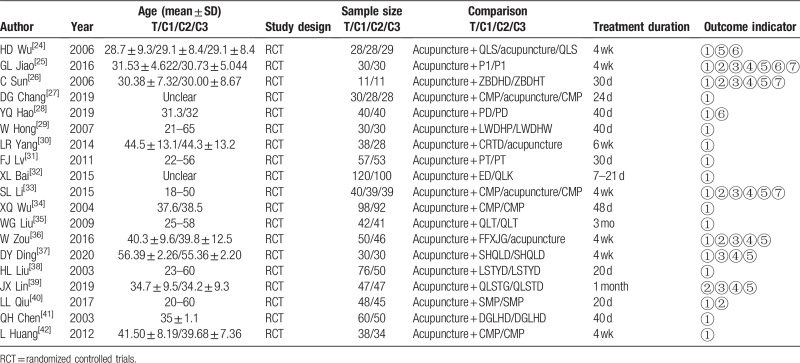
The basic characteristics of the included trials.

### 3.3 Quality evaluation results of included literature

**Figure 2:**
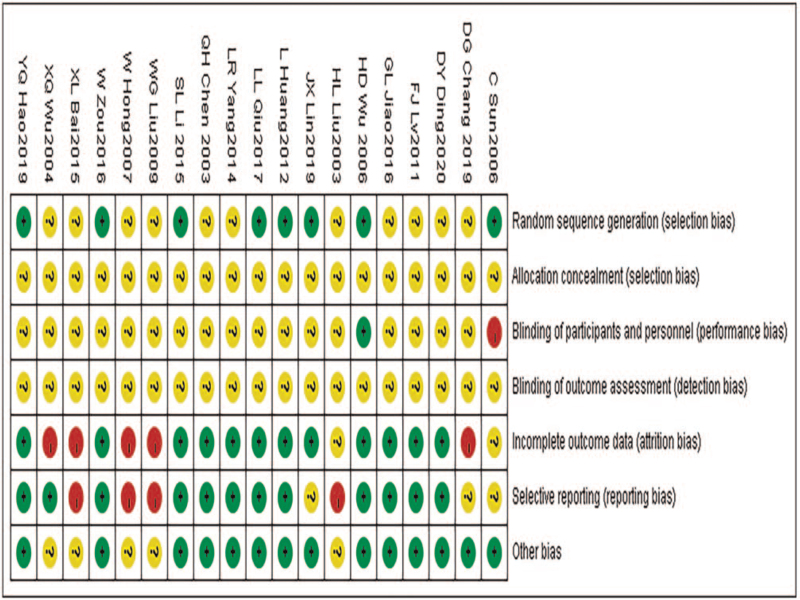
Summary of the risk of bias in the included studies.

In this study, 19 relevant RCT studies were included, with a total of 1831 cases. The risk of bias in the included literature was summarized in Figure 2.

### 3.4 Meta-analysis results

#### 3.4.1 Effective rate

**Figure 3:**
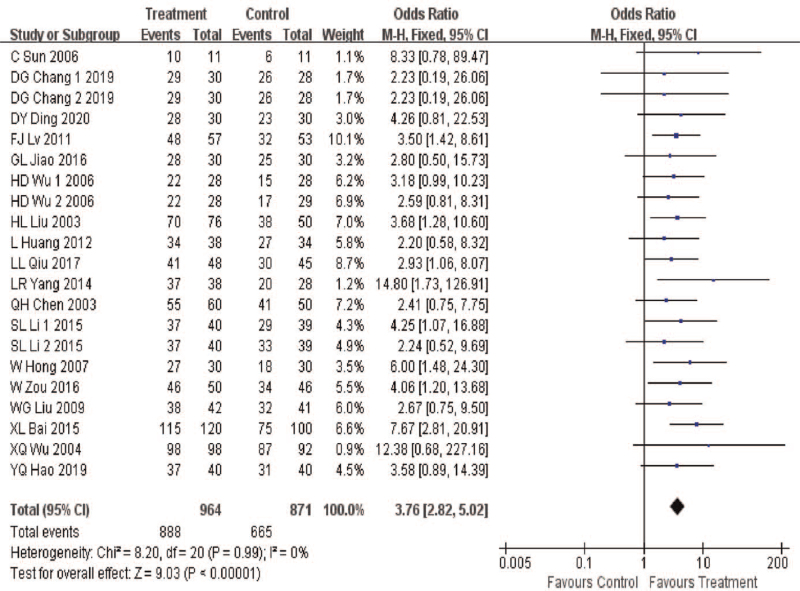
Efficient meta-analysis forest diagram.

A total of 21 pieces of literature were included, with a total of 1737 cases included. Heterogeneity test results: *P* = .99, *I*^2^ = 0%, indicating that there was no significant statistical difference in heterogeneity between studies. The fixed-effect model was used for analysis. Meta-analysis results showed that OR = 3.76, 95%CI (2.82, 5.02), *P* < .00001, indicating that the effective rate of the treatment group was 3.76 times that of the control group. It can be considered that the combination of acupuncture and TCM can significantly improve the clinical efficacy of CP, and the difference was statistically significant (*P* < .05), as shown in Figure 3.

#### 3.4.2 Total NIH-CPSI score

**Figure 4:**
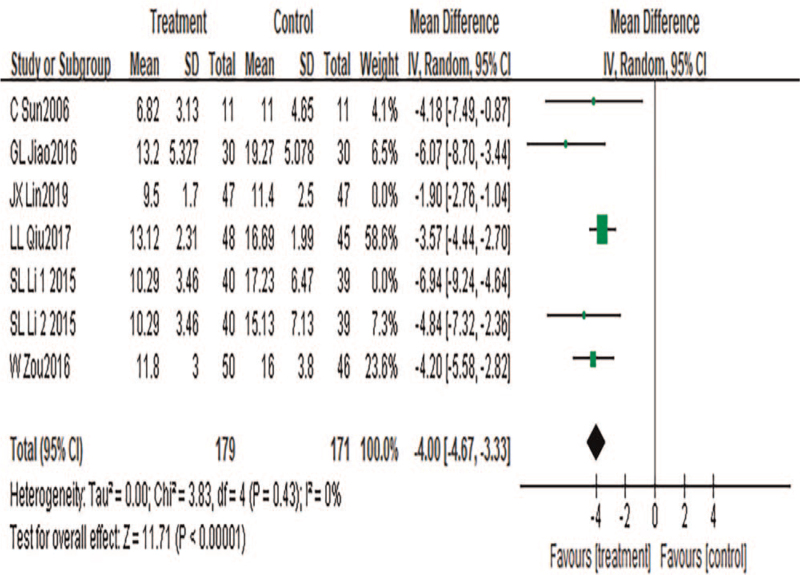
Meta-analysis forest diagram of NIH-CPSI total score.

A total of 7 pieces of literature were included, and a total of 523 cases were included. Heterogeneity test results: *P* = .0001, *I*^2^ = 78%. Sensitivity analysis was conducted to find the source of heterogeneity. The results showed that the heterogeneity disappeared when references [35,41] were excluded (*P* = .43, *I*^2^ = 0%). A fixed-effects model was used for meta-analysis. The results showed that the meta-analysis results were statistically significant (MD = −4.00, 95%CI: −4.67 to −3.33, *P* < .00001), suggesting that TCM combined with acupuncture could significantly improve the overall NIH-CPSI score for CP, as shown in Figure 4.

#### 3.4.3 NIH-CPSI urination score

**Figure 5:**
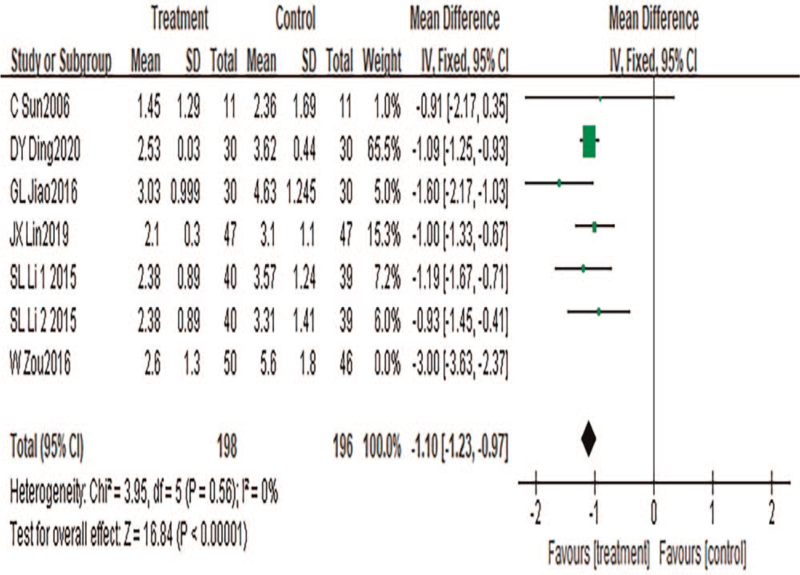
Meta-analysis forest diagram of NIH-CPSI urination score.

A total of 7 pieces of literature were included, with a total of 394 cases included. Heterogeneity test results: *P* < .00001, *I*^2^ = 84%. Sensitivity analysis was conducted to find the source of heterogeneity. The results showed that the heterogeneity disappeared when references [38] were excluded (*P* = .56, *I*^2^ = 0%). Further exploration revealed that the mean NIH-CPSI urination symptom score (8.0) in the pretreatment study [38] was significantly higher than the mean score (5.7) in other studies, suggesting that the heterogeneity was caused by the baseline effect of the included literature. A fixed-effects model was used for meta-analysis. Results showed that the results of the meta-analysis were statistically significant (MD = −1.10, 95%CI: −1.23 to −0.97, *P* < .00001), suggesting that TCM combined with acupuncture can reduce NIH-CPSI urination score and improve patients’ urination symptoms in patients with CP, as shown in Figure 5.

#### 3.4.4 NIH-CPSI pain score

**Figure 6:**
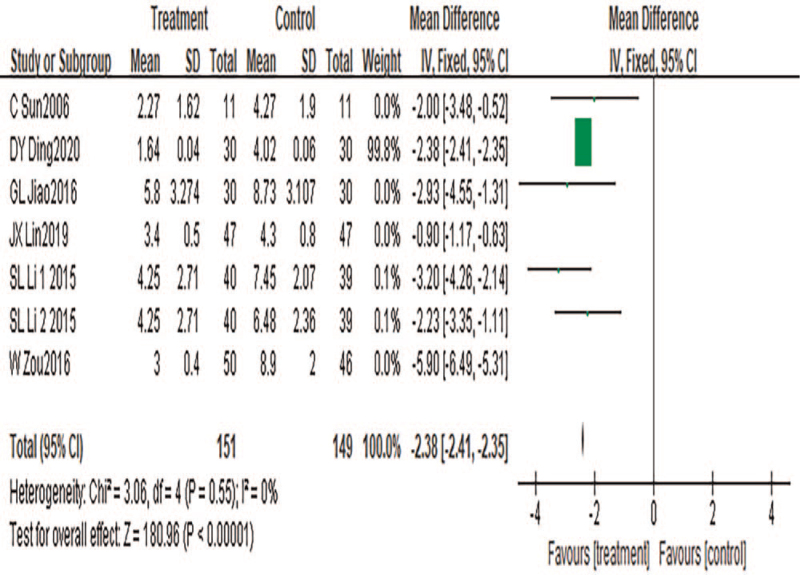
Meta-analysis forest plot of NIH-CPSI pain score.

A total of 7 pieces of literature were included, with a total of 300 cases included. Heterogeneity test results: *P* < .00001, *I*^2^ = 98%. Sensitivity analysis was conducted to find the source of heterogeneity. The results showed that the heterogeneity disappeared after the references [38,41] were excluded (*P* = .55, *I*^2^ = 0%). After an in-depth study of the literature, it was found that other literature only selected conventional acupuncture points for the treatment of CP during acupuncture treatment, while the treatment group of the literature [38,41] in addition to acupuncture conventional acupoints, also based on the patient's physique and condition are selected based on syndrome differentiation, and acupoints are appropriately matched to reduce the patient's NIH-CPSI pain score, reflecting the advantages of TCM syndrome differentiation. A fixed-effects model was used for meta-analysis. Results showed that the results of meta-analysis were statistically significant (MD = −2.38, 95%CI: −2.41 to −2.35, *P* < .00001), suggesting that TCM combined with acupuncture can significantly reduce NIH-CPSI pain score and relieve pain symptoms in patients with CP, as shown in Figure 6.

#### 3.4.5 NIH-CPSI quality of life score

**Figure 7:**
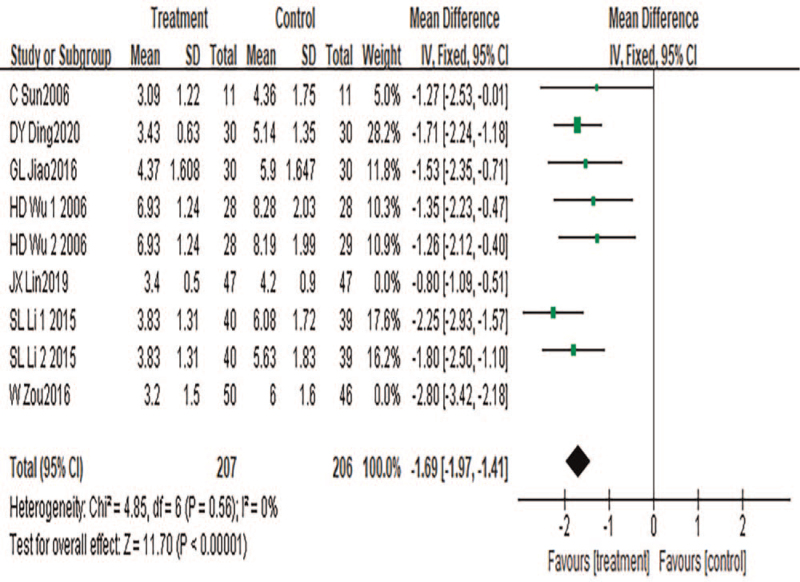
Meta-analysis forest diagram of NIH-CPSI quality of life score.

A total of 9 pieces of literature were included, with a total number of 413 cases included. Heterogeneity test results: *P* < .00001, *I*^2^ = 82%. Sensitivity analysis was conducted to find the source of heterogeneity. The results showed that the heterogeneity disappeared after the references^[36,39]^ were excluded (*P* = .56, *I*^2^ = 0%). Further study found that the heterogeneity was still caused by the acupoint selection based on syndrome differentiation in the treatment group,^[35,39]^ reflecting the importance of syndrome differentiation and acupuncture. A fixed-effects model was used for meta-analysis. Results showed that the results of the meta-analysis were statistically significant (MD = −1.69, 95%CI: −1.97 to −1.41, *P* < .00001), suggesting that Chinese medicine combined with acupuncture can reduce the NIH-CPSI quality of life score and improve the quality of life of patients with CP, as shown in Figure 7.

#### 3.4.6 TCM syndrome score

**Figure 8:**
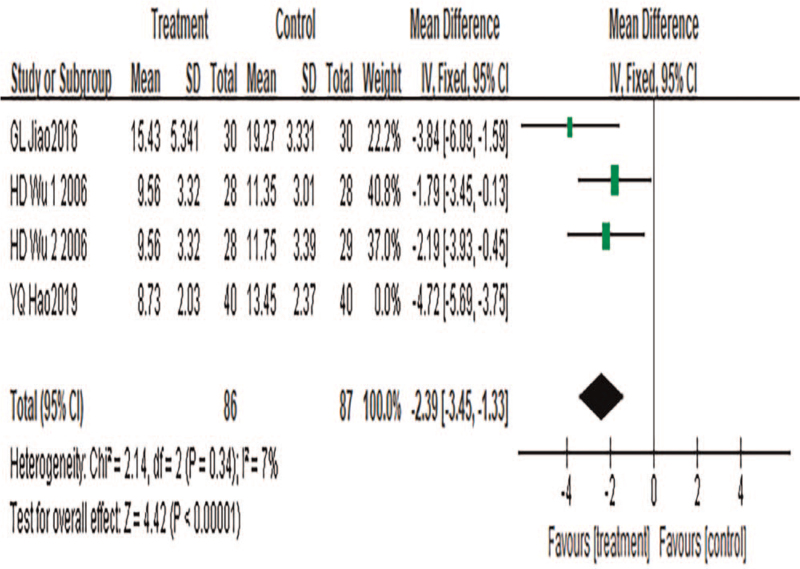
Meta-analysis forest diagram of TCM syndrome score. TCM = traditional Chinese medicine.

A total of 4 pieces of literature were included, with a total of 173 cases included. Heterogeneity test results: *P* = .007, *I*^2^ = 76%. Sensitivity analysis was conducted to find the source of heterogeneity. The results showed that the heterogeneity was significantly reduced after the exclusion of reference [30] (*P* = .34, *I*^2^ = 7%). Further studies found that only Zusanli was selected for acupuncture in the treatment group in the study,^[28]^ while acupoints were selected for acupuncture in other studies. Therefore, single acupoint selection can be considered as the source of heterogeneity. A fixed-effects model was used for meta-analysis. The results showed that the results of the meta-analysis were statistically significant (MD = −2.39, 95%CI: −3.45 to −1.33, *P* < .00001), suggesting that TCM combined with acupuncture can significantly reduce the scores of TCM symptoms in patients with CP, as shown in Figure 8.

#### 3.4.7 Adverse reactions

**Figure 9:**
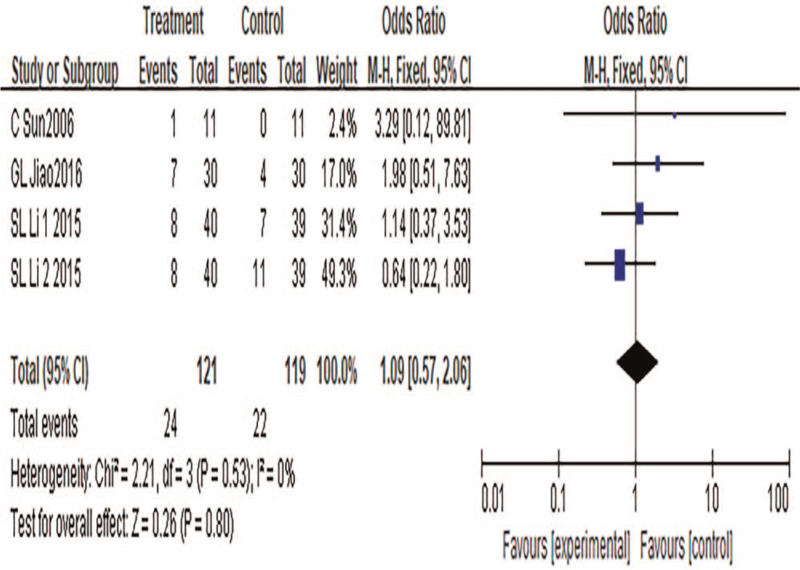
Meta-analysis forest diagram of adverse reactions.

A total of 4 articles were included, with a total number of 240 cases included. Heterogeneity test results: *P* = .53, *I*^2^ = 0%, suggesting no significant heterogeneity. A fixed-effect model was used for meta-analysis. Results showed that the results of the meta-analysis were not statistically significant (MD = 1.09, 95%CI: 0.57 to 2.06, *P* = .8), suggesting that there was no statistically significant difference in adverse reactions between the treatment group and the control group. TCM combined with acupuncture did not increase adverse reactions in patients with CP, and the safety was relatively high, as shown in Figure 9.

#### 3.4.8 Publication bias analysis

**Figure 10:**
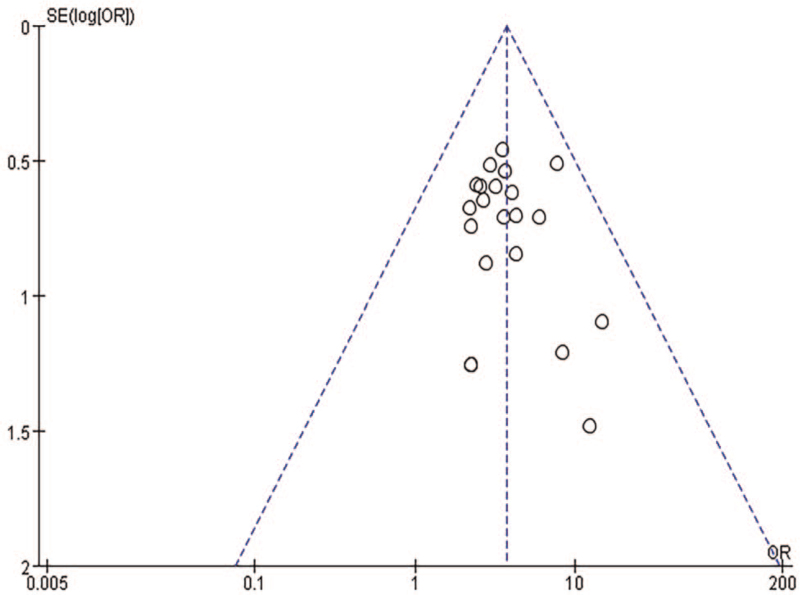
Funnel chart of efficient publication bias analysis.

Funnel plots were drawn using RevMan 5.3 software to evaluate publication bias. Funnel plots were drawn according to the efficiency, and the results showed that each point was on both sides of the axis of symmetry, presenting a symmetric funnel shape, suggesting that this study was less affected by publication bias and the conclusion was relatively reliable, as shown in Figure 10.

#### 3.4.9 Sequential analysis of efficiency tests

**Figure 11:**
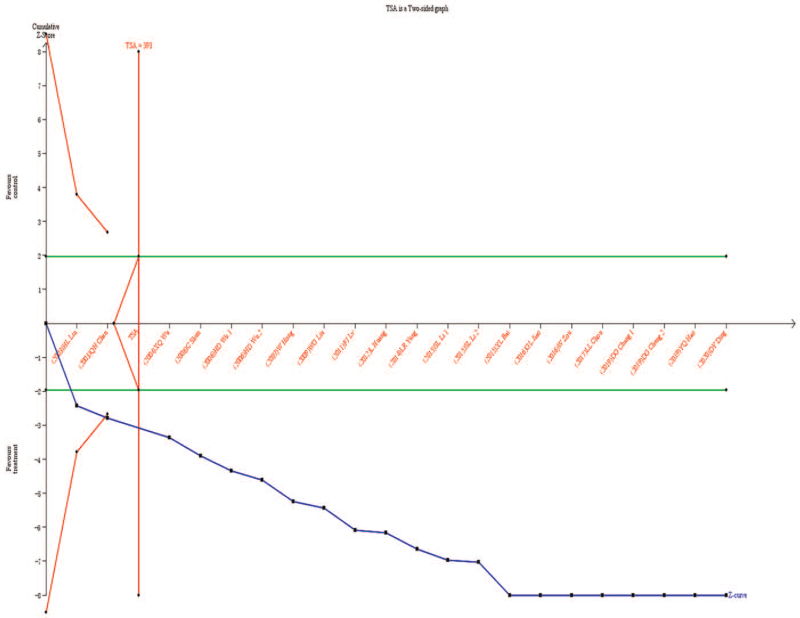
Efficient TSA results. TSA = test sequential analysis.

Using TSA v0.9 software, a single significance test with bilateral α = 5% was selected. Alpha value was set at 5%, the beta value was set at 20%, the statistical efficiency was 99.99%, the relative risk reduction rate was 0.01%, and the event rate of the control group was 76.35% (refer to the results of meta-analysis). TSA results show that the cumulative *Z* curve (blue curve) not only through the traditional boundary value (green line) the TSA boundary value (red curve), and incorporate the third study sample size has reached the expected information value (RIS) (vertical line), shows that Chinese medicine curative effect is combined acupuncture treatment of CP and meta-analysis of the positive results, as shown in Figure 11.

## 4 Discussion

### 4.1 Clinical efficacy and safety analysis

CP is one of the most common urinary disorders in men under 50 years of age. Its etiology is unknown, and there is no effective drug treatment. Due to the prostate's special anatomical position, its intima is deeply wrapped, which is easy to cause local microcirculation disturbance, blocked drainage, and difficult for therapeutic drugs to reach the lesion, which increases the difficulty of treatment.^[43]^ Prostatitis or prostate hyperplasia can coexist with prostate cancer. In a recent study,^[44]^ during the 12 months prior to the index date, 28% of the prostate cancer (CaP) patients had benign prostatic hyperplasia (BPH) and 2% had prostatitis. Prostate-specific antigen (PSA) is elevated in the most clinically significant CaP and is the most important early detection indicator. Although PSA is the most commonly used test for CaP, BPH and prostatitis can also produce PSA-positive results. One way to solve this problem is to check for PSA. Regular check-ups can help diagnose CaP. Chronic prostatitis is an important cause of elevated PSA. When PSA is elevated, we should actively treat it. Treatment of CP may reduce PSA.^[45]^ Elevated PSA is a risk factor, we should eliminate this risk factor through treatment, reduce its occurrence of CP, BPH, and CaP.

Current studies have shown that drug interventions can improve the overall NIH-CPSI score and, to some extent, improve most symptoms in patients with CP/CPPS, but no single drug can completely cure all symptoms in patients with CP.^[46–47]^ Acupuncture and TCM, provide a new direction for treating this disease. This is the first meta-analysis to explore the safety and efficacy of acupuncture combined with TCM in CP treatment. The results showed that acupuncture combined with TCM can significantly improve the symptoms of urination, relieve pain, and improve the quality of life of patients with prostatitis, and it does not increase the incidence of adverse reactions. Acupuncture as a part of TCM is widely accepted in the clinic because of its safety and obvious efficacy. Acupuncture treatment of chronic prostatitis main acupoints include Zhongji, Guanyuan, Shenshu, Sanyinjiao, and Taichong. These acupuncture points have been used in many studies on the treatment of chronic prostatitis. Clinically, acupuncture combined with TCM is used to treat CP. It is recommended to apply for acupuncture and medicine according to syndrome differentiation. Acupuncture and TCM are targeted according to the patient's condition and constitution. Acupoint selection based on conventional acupuncture can relieve pain and improve the quality of life of patients. Acupuncture can direct qi to the clinic, which has the advantage of targeted treatment of TCM. On the other hand, adjusts the body based on the holistic concept and provides the macroscopic treatment. The combination of acupuncture and TCM, reflecting the characteristics of TCM syndrome differentiation treatment, can give full play to the comprehensive advantages and promote the overall improvement of clinical efficacy. At the same time, it also has a positive effect on the rehabilitation of patients.

### 4.2 Shortcomings of this meta-analysis

Acupuncture, as a part of TCM, has been used in many countries. In contrast, TCM has not been recognized abroad due to the complexity of its composition and mechanism of action, but its efficacy cannot be ignored. At present, the data of foreign application of acupuncture combined with TCM in the treatment of CP cannot be widely obtained. This study was included in the research literature. The subjects of the study were mainly Chinese residents. Regional and ethnic restrictions may affect the overall objectivity of this meta-analysis. As TCM's theoretical system is mainly characterized by the holistic concept and syndrome differentiation, comprehensive conditioning of patients’ condition is mainly adopted at the macro level. In addition to the main acupoints of the disease, different acupuncture points selection and acupuncture techniques will be added according to different patients in the treatment, which cannot achieve complete unity of all aspects. The TCM and patent Chinese medicine selected for combination therapy are also different, which is also one of the reasons for its heterogeneity.

## 5 Conclusion

In conclusion, acupuncture combined with TCM is effective and safe in the treatment of CP. The results obtained in this study could potentially be applied to existing practical evidence-based guidelines for the treatment of CP. Nevertheless, further investigation and long-term follow-up are crucial to further clarify the long-term efficacy of acupuncture combined with TCM.

## Author contributions

**Conceptualization:** Guozheng Qin.

**Data curation:** Chenxi Li, Lei Xu, Xuyao Lin, Lichao Li.

**Formal analysis:** Chenxi Li, Lanlan Li, Yue Zhang.

**Methodology:** Chenxi Li, Lei Xu, Hua Li.

**Resources:** Xuyao Lin, Qingrui Li, Mingkai Wang.

**Software:** Chenxi Li, Pule Ye, Lin Wu.

**Supervision:** Guozheng Qin.

**Writing – original draft:** Chenxi Li, Lei Xu, Qingrui Li.

**Writing – review & editing:** Guozheng Qin, Chenxi Li.
